# Central precocious puberty during COVID-19 pandemic and sleep disturbance: an exploratory study

**DOI:** 10.1186/s13052-022-01256-z

**Published:** 2022-04-23

**Authors:** Giuseppina R Umano, Ivan Maddaluno, Simona Riccio, Francesca Lanzaro, Rachele Antignani, Maria Giuliano, Caterina Luongo, Adalgisa Festa, Emanuele Miraglia del Giudice, Anna Grandone

**Affiliations:** 1 Department of the Woman, the Child, of General and Specialized Surgery, University of Campania “L. Vanvitelli”, Naples, Italy; 2 Società Italiana Medici Pediatri Campania (SIMPe), Teramo, Italy

**Keywords:** Central precocious puberty, COVID-19, Sleep, Children

## Abstract

**Background:**

Increased incidence of central precocious puberty (CPP) after coronavirus infectious disease-19 lockdown has been reported. Our study aims in investigating changes in CPP rates and in sleep patterns in CPP and healthy controls.

**Methods:**

CPP were retrospectively evaluated from April 2020 to April 2021. Parents of girls diagnosed with CPP during lockdown and of matched healthy controls filled out a questionnaire about sleep disturbances (SDSC questionnaire) and sleep schedules.

**Results:**

Thirty-five CPP and 37 controls completed the survey. Incidence of new CPP cases significantly increased in 2020–2021 compared to 2017–2020 (5:100 vs 2:100, *p* = 0.02). Sleep disturbance rates did not differ between CPP and healthy controls before lockdown. During lockdown, CPP reported higher rates of sleep disturbs for total score (*p* = 0.005), excessive somnolence (*p* = 0.049), sleep breathing disorders (*p* = 0.049), and sleep–wake transition disorders (*p* = 0.005). Moreover, CPP group more frequently shifted toward later bedtime (*p* = 0.03) during lockdown compared to controls. Hours of sleep and smartphone exposure around bedtime did not differ between groups.

**Conclusions:**

Our study confirms the observation of increased incidence of CPP after lockdown measures. Additionally, CPP showed higher rates of sleep disturbances and later bedtime compared to controls. The causality link between sleep disturbances and CPP should be further investigated to gain knowledge in this association.

## Background

On January 2020, a new severe acute respiratory syndrome (SARS) due to a previously unknown coronavirus infection was firstly reported in China [1]. Since then, the diffusion of the so-called COVID-19 (coronavirus infectious disease-19) has reached pandemic proportion.

With the aim of containing the spread of COVID-19, several Governments have imposed restrictive policies promoting social isolation and “stay at home”. National lockdowns lead to sudden and radical changes in social interactions and in study and working conditions. Italian government declared national lockdown on March 10^th^, 2020, since then people were forced to stay at home, and children dramatically changed their lifestyle for several months. In addition to national closures, Campania region imposed more severe restrictions and longer lockdown periods (March 2020 to May 2020 and November 2020 to April 2021). Given that, significant changes in lifestyle habits have been reported in children and adolescents. It has been registered an increase in sedentary behavior, junk food and sugar sweetened beverage intake, and sleep–wake rhythm dysregulation [2, 3]. Moreover, during the last months, scientific evidence has been produced about indirect consequences of lock-down and lifestyle habits modifications on children’s wellbeing. A significant and rapid raise of precocious puberty has been reported in Italy compared to past years [4, 5]. Puberty is known to be a complex phenomenon that is influenced by genetic, metabolic, psychological, and environmental factors. The mechanisms underlying puberty timing have not been completely clarified as several hormones and systems are involved [6]. Among them, melatonin has been proposed as one of neuromodulator of puberty onset [7–9]. Melatonin is secreted by pineal gland, and it is mainly involved in sleep–wake cycle regulation. However, it also influences hypothalamic-pituitary–gonadal axis maturation [10].

Based on this evidence, our study aims were: 1- to evaluate the changes in precocious puberty rates during lockdown in our tertiary centre of paediatric endocrinology in South Italy (where confinement measures and school closure were more severe compared to other Italian regions); 2- to investigate the differences in sleep habits and disturbances in girls with central precocious puberty compared to healthy controls.

## Methods

### Subjects

We prospectively enrolled female patients that attended the outpatient clinic of paediatric endocrinology of the University of Campania Luigi Vanvitelli because of central precocious puberty (CPP) during the lockdown period (April 2020 to April 2021). Their clinical and biochemical data and CPP incidence were compared to those of the previous 3 years (2017–2020). CPP was defined as breast development before 8 years of age and by central hypothalamic–pituitary–gonadal activation identified by pubertal basal luteinizing hormone (LH) levels (LH > 0.3 UI/L) and/or GnRH-stimulated (0.1 mg Relefact LHRH; Sanofi-Aventis, Frankfurt am Main, Germany) LH levels > 5 IU/L [11, 12]. Magnetic resonance imaging of the central nervous system was normal in all patients. Additionally, we enrolled matched prepubertal healthy girls as control group. Control subjects were recruited from outpatient clinics for auxological evaluation in the same period and selected if growth parameters were in the normal range for age and sex. Girls with chronic diseases and assuming drugs affecting sleep/wake rhythm were excluded. Clinical examination was performed in all girls, including weight and height measurement, z-score BMI calculation using the LMS method and staging of breast development according to Tanner’s classification [13, 14].

The study was conducted in accordance with the Declaration of Helsinki. The protocol was approved by the ethical committee of the University of Campania (Protocol number 23.26–20,200,008,943), and informed consent was obtained from the parents or guardians.

### Diagnostic work-up

Chemiluminescence assay (LIAISON, Diasorin) was used to measure Follicle Stimulating Hormone (FSH) and LH concentrations, with detection limits of 0.06 and 0.05 U/L, respectively, and intra- and inter-assay CV less than 5%. Radioimmunoassay was used to measure serum estradiol (CisBio International). The analytical and functional detection limits for plasma estradiol were 4 and 8 pg/mL, respectively.

GnRH stimulation test was provided for patients in which basal hormone level did not meet diagnostic criteria for CPP. Peak-LH > 5UI/L after administration of 0.1 mg of Relefact LH-RH (Sanofi-Aventis, Frankfurt am Main, Germany) was considered positive.

Bone age was estimated according to Tanner-Whitehouse 2 (TW2) method.

Thin-section, contrast-enhanced MRI examination of sellar region with T1-weighted and T2 weighted sagittal, coronal sequences, 3DT2 thin section axial sequence and FLAIR and EPI DWI on axial sequence was acquired for all patients.

### Sleep habits measures

Sleep disturbs were investigated by SDSC (sleep disturbance scale for children) questionnaire. This questionnaire has been validated in Italian school aged children and adolescents by Bruni et al. in 1996 [15]. The questionnaire comprehends 26 items scored in a 5-point Likert scale according to disturb frequency. The 26 items could be grouped in six subscales, namely: disorders of initiating and maintaining sleep (DIMS); sleep breathing disorders (SBD); disorders of arousal (DOA); sleep wake transition disorders (SWTD); disorders of excessive somnolence (DES), and sleep hyperhidrosis (SH). Moreover, a total score of global disturb might be obtained by the sum of subscales scores. Recently, several studies have investigated sleep disturbs in children during lockdown by SDSC questionnaire [3, 16–19]. In addition to SDSC, we registered bedtime, hours of sleep, and use of smartphone around bedtime, use of electronic devices during the day and for e-learning was not investigated. Bedtime was grouped as: before 10.00 pm; 10.00–11.00 pm; 11.00 pm-00.00am; and after 00.00am. Caregivers were asked to answer the questionnaire about children sleep habits. Questionnaires were administered during lockdown period. In addition, they filled out a second questionnaire referred to sleep behavior before lockdown with the aim of investigate changes in behaviors between the two periods.

### Statistical analysis

Continuous variables were checked for normality distribution by Shapiro–Wilk test. Differences in continuous variables were investigated by Student t-test and Mann–Whitney U test as appropriate. Differences in categorical variable were evaluated by Fisher exact test and Chi square test as appropriate. SDSC scores were defined as pathologic score if scores were total ≥ 71, DIMS ≥ 17, SBD ≥ 7, DOA ≥ 6, SWTD ≥ 14, DES ≥ 13, and SH ≥ 7 [15]. CPP incidence was calculated as the ratio of CPP diagnosis and number of outpatients visits a year. Continuous data are expressed as mean ± standard deviations (DS). Categorical data are reported as frequency. A *p* value < 0.05 was considered statistically significant. All analyses were performed with SAS University Edition software.

## Results

### Central precocious puberty incidence

Thirty-five girls with CPP attended our endocrinologic outpatient clinic from April 2020 to April 2021. Retrospective survey revealed that during the previous three years we observed 34 girls with CPP (average of 11 case/year). The overall number of new cases in 2020–2021 was higher than those observed in 2017–2020. Moreover, CPP incidence rate was 2.5-fold higher in 2020–2021 (5:100) compared to 2017–2020 (2:100, *p* = 0.002). Characteristics of CPP diagnosed before lockdown and after/during lockdown are reported in Table 1. The two groups did not differ for age, DS-height, z-score BMI, familial history of precocious puberty, and peak-LH levels (Table 1). Conversely, we found significant higher levels of LH, FSH, and 17-beta estradiol in CPP after/during lockdown compared to those diagnosed before (Table 1).

**Table 1:** Clinical and biochemical characteristics of CPP cases diagnosed before and after lockdown measures

	CPP before lockdown (*N* = 34)	CPP during lockdown (*N* = 35)	P
Age (ys)	7.97 ± 1.11	7.59 ± 0.67	0.10
Height (cm)	129.25 ± 6.99	129.69 ± 5.87	0.66
DS-Height	0.17 ± 1.04	0.78 ± 1.0	0.05
Z-score BMI	0.60 ± 0.66	0.23 ± 1.51	0.31
Bone age (ys)	9.33 ± 1.12	8.77 ± 0.67	0.19
LH (UI/ml)	0.87 ± 0.79	1.23 ± 1.12	**0.03**
FSH (UI/ml)	4.34 ± 2.43	7.09 ± 2.47	**0.02**
Peak LH (UI/ml)	8.93 ± 4.35	8.05 ± 2.47	0.60
17-beta estradiol (pg/ml)	19.38 ± 14.23	30.88 ± 22.53	**0.04**
Positive familial history (%)	21.4	46.4	0.18

*BMI* body mass index, *FSH* follicle-stimulating hormone, *LH* Luteinizing hormone

Data are expressed as mean ± standard deviations or as frequencies

### Central precocious puberty and sleep measures

A total of 72 parents (35 CPP and 37 controls) completed the survey. Girls with CPP were taller than controls (*p* = 0.002), whereas there were no differences for age (7.8 ± 0.7 versus 7.6 ± 0.9, *p* = 0.10), BMI (18.6 ± 3.3 versus 17.7 ± 3.6, *p* = 0.20), z-score BMI (1.1 ± 1.3 versus 0.7 ± 1.4, *p* = 0.22), and overweigh and obesity prevalence (34% versus 25%, *p* = 0.41) between groups.

**Fig. 1:**
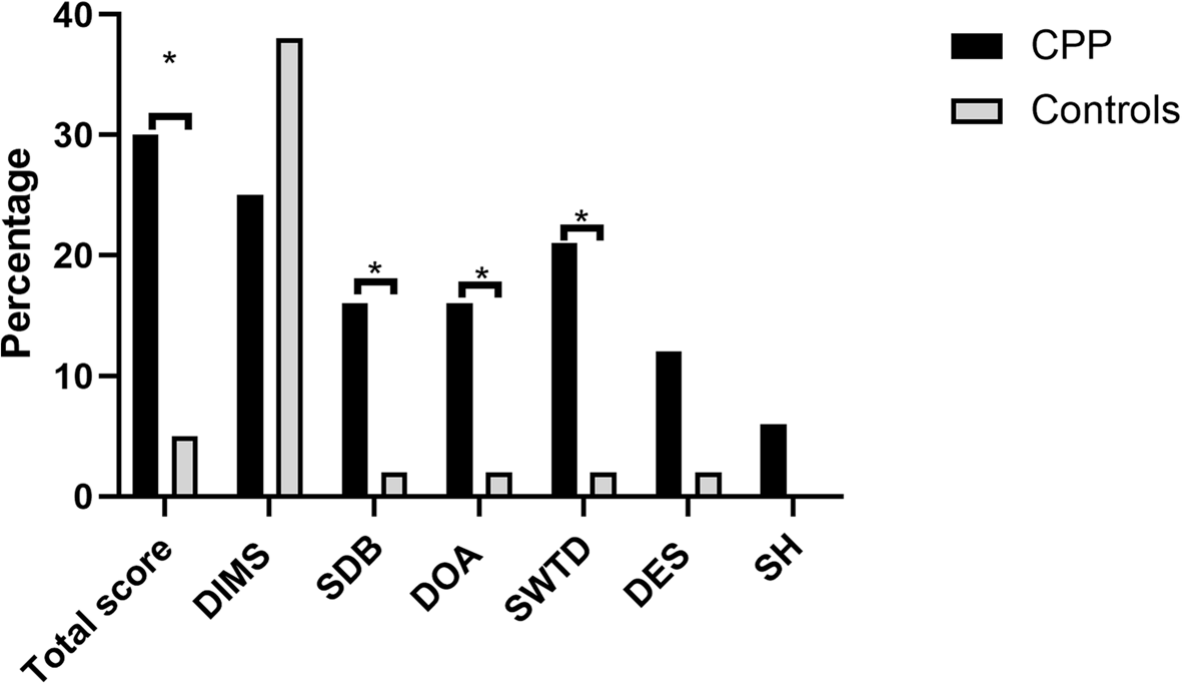
Distribution of pathologic scores according to groups. Star indicates significant differences. Legend: DIMS: disorders of initiating and maintaining sleep; SBD: sleep breathing disorders; DOA: disorders of arousal; SWTD: sleep wake transition disorders; DES: disorders of excessive somnolence; SH: sleep hyperhidrosis

Rates of pathologic SDSC scores did not significantly differ between the two groups for questionnaires referred to the period before lockdown. Conversely, we observed significant higher rates of altered score calculated for questionnaires during lockdown in CPP group compared to controls for total score (31.4% versus 5.3%, *p* = 0.005), DES (17.1% versus 2.6%, *p* = 0.049), SBD (17.1% versus 2.6%, *p* = 0.049), and SWTD (25.7% versus 2.63, *p* = 0.005). No differences were found for disorders in initiating and maintaining sleep (DIMS), disorders of arousal (DOA), and sleep hyperidrosis (SH) (see Fig. 1).

With regards to bedtime, CPP group showed significant higher rates in shifting toward later bedtime during lockdown compared to controls (40.3% vs 17.7%, respectively, *p* = 0.03). However, total sleep hours did not significantly change before and during lockdown in both CPP and controls (*p* = 0.10 and *p* = 0.11, respectively). We also investigated electronic devices time exposure around bedtime and found no differences between the two groups (*p* = 0.23).

## Discussion

Our study confirms previous reports of increased CPP incidence rates during and after lockdown for COVID-19 in Italy [4, 5]. In our population, disease incidence more than doubled compared to the previous 3 years. Additionally, we found increased oestradiol, LH, and FSH levels in girls diagnosed after lockdown compared to previous years. This finding is in line to those reported by Stagi et al. that observed an acceleration of puberty progression in cases diagnosed after lockdown compared to the previous 5 years [4].

It is well known that puberty timing is influenced by both environmental and genetic factors. The dramatical changes in everyday routine that characterized lockdown period might have been a powerful trigger for puberty onset. However, the underlying mechanisms have not been elucidated and several hypotheses have been proposed, such as weight gain, psychological stress, and electronic devices overuse. In our sample we found no differences for electronic device use and adiposity measures between CPP and controls. Conversely, we observed higher prevalence of sleep disturbance and shift in later bedtime in CPP compared to control group. Main differences were observed for total disturbs score, sleep breathing disorders, sleep–wake transition disorders, and daily excessive somnolence.

The directionality of this association needs to be clarified. In fact, sexual steroids have been associated with changes in circadian rhythm in animal studies reporting later phase shift of daily rhythms in animals [20, 21]. However, these data have not been replicated in humans. Jessen et al. reported a correlation between 17OH-progesterone plasma level and bedtime in girls with premature pubarche. Nevertheless, no significant association were observed in girls with CPP [22]. Another study investigating the effect of oestrogens on sleep architecture in girls with CPP, failed to find any difference in sleep patterns in this group of patients before and after hormonal therapy, suggesting that oestrogens are not involved in sleep architecture regulation in this group of patients [23]. Therefore, more studies are needed to investigate whether sexual hormones might affect sleep homeostasis in humans, especially during puberty.

At the same time, it has been reported that changes in sleep hormones might influence puberty timing. In fact, melatonin plasma levels decrease has been associated with puberty onset in children [10, 24]. A study comparing melatonin plasma levels between precocious puberty (PP), normal puberty, and pre-pubertal girls, revealed that girls with PP had significantly lower melatonin levels compared to the other groups [25]. Whether melatonin directly inhibits the hypothalamus or reduces the pituitary response to GnRH should be further investigated. However, melatonin plasma levels physiologically decrease throughout growth; therefore, this reduction might allow the activation of the hypothalamus-pituitary-gland axis [24]. In our sample, girls with CPP showed higher rates of SBD and SWTD compared to controls. Sleep disturbances have been associated with lower levels of melatonin secretion and with melatonin secretion pattern disruption [26–28]. Therefore, it might be hypothesized that CPP group experienced a more severe melatonin plasma decrease compared to controls that could have acted as puberty trigger. Additionally, the age of this group is close to physiologic puberty onset, therefore melatonin reduction might enable the activity of an almost mature hypothalamic-pituitary-gonad axis [29].

On the other hand, sleep homeostasis is essential for cognitive and emotional development [30]. In fact, sleep disturbances have been associated with poor quality of life, impairment of everyday functioning, increased rate of mood and behavioral disorders [31–33] and impairment of academic performance [34–36]. Therefore, it is very important to recognize and treat sleep disruption. Considering our findings, girls with CPP might constitute a group of patients at higher risk of presenting sleep disorders and clinicians should be aware of this association and investigate the presence of sleep disturbances in this subgroup of patients. Therefore, we plan to extend our investigation to new CPP diagnosis even outside lockdown period aiming to eventually confirm this observation. Additionally, our exploratory findings might constitute the basis for new longitudinal studies investigating the mechanisms underlying the relationship between puberty and sleep homeostasis in larger cohorts.

## Conclusions

In conclusion our study supports the observation of increased incidence of CPP after lockdown. Home confinement has significantly altered daily routine in children and adolescents and indirect health consequence of confinement are arising. In our sample, sleep disturbs are a frequent comorbidity in girls with CPP, and clinicians should be aware of this association. Further pathophysiologic studies are needed to investigate whether sleep alteration might be a trigger for puberty onset.

## Data Availability

The datasets used and/or analyzed during the current study are available from the corresponding author on reasonable request.

## Abbreviations

COVID-19: Coronavirus Infectious Disease-19
CPP: Central Precocious Puberty
DES: Disorders of Excessive Somnolence
DIMS: Disorders of Initiating and Maintaining Sleep
DOA: Disorders of Arousal
FSH: Follicle Stimulating Hormone
LH: Luteinizing Hormone
SARS: Severe Acute Respiratory Syndrome
SBD: Sleep Breathing Disorders
SDSC: Sleep Disturbance Scale for Children
SH: Sleep Hyperhidrosis
SWTD: Sleep Wake Transition Disorders
